# The Influence of *t*-Butyl and Cyclododecyl Substitution on Ethylene/1-Hexene Copolymerization Using Ansa-Fluorenylamidodimethyltitanium Derivatives

**DOI:** 10.3390/molecules16054122

**Published:** 2011-05-19

**Authors:** Patcharaporn Kaivalchatchawal, Piyasan Praserthdam, Yuuichi Sogo, Zhengguo Cai, Takeshi Shiono, Bunjerd Jongsomjit

**Affiliations:** 1 Center of Excellence on Catalysis and Catalytic Reaction Engineering, Department of Chemical Engineering, Faculty of Engineering, Chulalongkorn University, Bangkok 10330, Thailand; 2 Department of Applied Chemistry, Graduate School of Engineering, Hiroshima University, Higashi-Hiroshima 739-8527, Japan

**Keywords:** polymer synthesis, catalytic synthesis, polyolefins, metallocene catalyst, CGC catalyst

## Abstract

In the present study, copolymerization of ethylene and 1-hexene was conducted with a series of ansa-fluorenylamidodimethyltitanium complexes, including [*t*-BuNSiMe_2_Flu]TiMe_2_ (complex **1**), [cyclododecylNSiMe_2_Flu]TiMe_2_ (complex **2**) and [*t*-BuNSiMe_2_(2,7-t-Bu_2_Flu)]TiMe_2_ (complex **3**), activated by MMAO. The effect of these catalysts on catalytic behavior, namely activity, molecular weight and monomer reactivity ratios, has been investigated. The results showed that all of them acted by a single site polymerization mechanism and the molecular weight distribution is independent of catalyst structure. Based on the study, it revealed that the introduction of a *t*-butyl at the 2,7 position on the fluorenyl ligand is able to enhance both catalytic activity and copolymer molecular weight more than introducing a cyclododecyl on the amine, which is probably associated with the electronic effect exerted by the *t*-butyl substituent. The comonomer incorporation content was controllable over a wide range by adjusting the comonomer feed ratio. Moreover, referring to monomer reactivity ratio exploration, it seems that the substitution on the ansa-fluorenylamidodimethyltitanium complex tends to hinder the insertion of 1-hexene into the polymer chain, leading to the highest 1-hexene content for traditional complex **1**.

## 1. Introduction

At present, linear low density polyethylene (LLDPE) is regarded as an important type of polyethylene and is also well recognized as economically attractive, accounting for more than half of the annual worldwide polymer production due to its distinctive processing and mechanical properties [1]. Copolymerization of ethylene and α-olefins, such as 1-butene, 1-hexene and 1-octene, is a general way to generate LLDPE with short chain branching. This copolymerization usually involves a constrained geometry catalyst (CGC) which has opened active sites for easy insertion of high α-olefins [2,3,4,5]. Superior to conventional Zeigler-Natta and metallocene catalysts, CGCs are capable of improving products in terms of much higher comonomer incorporation, narrower comonomer and molecular weight distribution which leads to better mechanical and physical properties. However, the incorporation of α-olefins that would result in the polymer properties depends on the structure of catalyst employed during copolymerization [1,6,7]. In fact, small variations of the ligand structure or ligand substituents may cause profound changes in the catalytic activity, copolymerization behavior and properties of the resulting polymer [3,4,5,8]. Therefore, by knowing the nature of catalysts, properties can be controlled and altered in order to achieve the desired LLDPE.

**Scheme 1:**
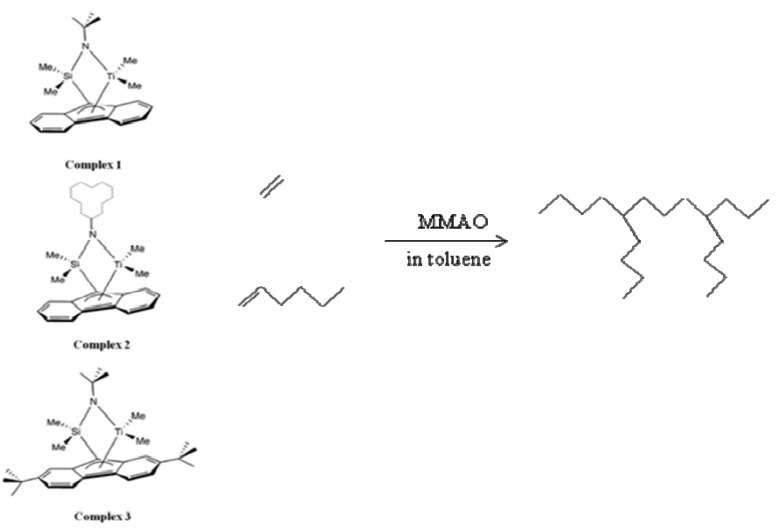
Ethylene/1-hexene copolymerization system.

In this research, complexes **1**, **2** and **3** (Scheme 1) were synthesized and further used as the catalysts for ethylene/1-hexene copolymerization to investigate the effect of the CGC-titanium complex on the copolymerization behavior.

## 2. Results and Discussion

The ethylene/1-hexene copolymerization with catalyst complexes **1**, **2** and **3** was carried out at 273 and 298 K under atmospheric pressure whereas the polymerization time of each batch was adjusted so as to keep the comonomer conversion relatively constant for the purpose of acceptable reactivity ratio evaluation. The catalytic results are tabulated in Table 1.

**Table 1:** Summary of ethylene/1-hexene copolymerization catalytic activities and properties of copolymers.

Entry ^a)^	1-hexenemol^.^L^−1^	Time s	Yield g	Activity ^b)^ kg^.^mol^−1 ^Ti^.^h^−1^	cont ^c) ^%	Mw ^d) ^kg^.^mol^−1^	MWD ^e)^
T	0.75	60	0.6832	2050	-	-	-
1-1	0.075	210	0.0265	23	37.2	27	1.44
1-2	0.15	240	0.0835	63	37.8	25	1.53
1-3	0.45	360	0.2477	122	62.1	44	1.73
1-4	0.75	200	0.3688	221	74.3	49	1.53
1-5	1.5	180	0.3691	369	72.1	60	1.51
2-1	0.075	180	0.0813	81	30.1	26	1.61
2-2	0.15	120	0.1514	227	40.7	35	1.47
2-3	0.45	120	0.2250	338	57.9	36	1.54
2-4	0.75	150	0.3399	408	66.9	38	1.58
2-5	1.5	180	0.6160	616	78.0	71	1.48
3-1	0.075	45	0.0856	342	33.8	37	1.7
3-2	0.15	45	0.1167	467	47.2	64	1.48
3-3	0.45	40	0.2089	940	64.1	166	1.83
3-4	0.75	35	0.5272	2711	63.0	329	1.70
3-5	1.5	30	0.6006	3604	68.3	415	1.50

^a)^ Entry T, 1-1, 1-2, 1-3, 1-4 and 1-5 used complex 1 as catalyst; Entry 2-1, 2-2, 2-3, 2-4 and 2-5 used complex 2 as catalyst; Entry 3-1, 3-2, 3-3, 3-4 and 3-5 used complex 3 as catalyst; ^b)^ polymerization condition: [Ti] = 20 µmol, MMAO as cocatalyst, [Al]/[Ti] = 400, liquid volume (toluene) = 30 mL, ethylene pressure = 1 atm, temperature = 273 K (except Entry T (293 K)); ^c)^ 1-hexene content in copolymer determined by ^13^C NMR; ^d)^molecular weight determined by GPC using PS standard; ^e)^molecular weight distribution (Mw/Mn) determined by GPC using PS standard.

Regarding the result of Entry T in Table 1, it can be seen that the activity of the complex **1** when the reaction was performed at 293 K was very high (2,050 kg^.^mol^−1^ Ti^.^h^−1^), leading to difficulty in controlling the comonomer conversion. As a consequence, the other polymerization temperature conditions were reduced to 273 K. For comparison of the three sets of catalytic systems, including using catalyst complexes **1**, **2** and **3**, the activity towards ethylene/1-hexene was in the order complex **3** > complex **2** > complex **1**. In detail, the attachment of *t*-butyl groups at the 2,7 positions of the fluorenyl ring significantly impacted the catalytic behavior of the CGC complex, causing about 7–10 times higher activity than that obtained from the original nonsubstituted complex **1**. This was in good agreement with the previous research on propylene polymerization under the specified polymerization conditions [9]. It also showed that this increased catalytic activity is presumably due to an enhancement of the propagation rate by the electronic effect of the alkyl groups. The introduction of cyclododecyl on the amine group also resulted in an increase of activity, but less pronounced. 

Likewise, a similar trend in copolymerization activity for these complexes can be observed. As the 1-hexene feed concentration rose, the catalytic activity increased. Relating to Table 1, it is fair to say that the activity displayed by complex **3** was more sensitive to the comonomer concentration. This phenomenon has been generally known as “comonomer effect” and has been described in a large number of reports [2,10]. The rate-time profiles of copolymerization demonstrated that all profiles were similar, starting with the minimum initial value and then gradually increasing with time, proposing that no deactivation of catalyst occurred during the copolymerization.

The relevant GPC results of obtained copolymers are collected in Table 1. All of resultant polymers possessed middle to high molecular weight and unimodal molecular weight distribution (Mw/Mn < 2), conforming the single site polymerization behavior of the three complexes. Nevertheless, it is noticeable that the molecular weight of copolymer obtained with complex **3** was 3–5 times higher than that of the corresponding copolymers obtained with the remaining complexes. Therefore, we may concluded that the 2,7 *t*-butyl substituent has a profound effect on a molecular weight increase. This is probably ascribable to the fact that the electronic effect exerted by the substituent reduces the rate of chain termination. Considering the relationship between the molecular weight and comonomer feed concentration, the molecular weight of all copolymers produced by the three titanium complexes increased with the rise of concentration, contrary to the literature [2]. This trend was rather unexpected since comonomer incorporation usually favors chain termination on account of terminal double bonds formed by hydride β-elimination which are mainly between comonomer units, consequently causing lower molecular weight copolymers.

A quantitative analysis of triad distribution was carrried out using ^13^C-NMR spectra assignment [11] of ethylene/1-hexene copolymer and is shown in Table 2. As expected, for each catalyst complex employed, the incorporation of comonomer increased with an increase in 1-hexene concentration in the reaction medium. Even though the content was slightly dependent on the complex used, all CGC complexes yielded the copolymers with high 1-hexene content (>30% mol). 

The best way to investigate a copolymerization is to measure the reactivity ratios of the ethylene monomer (**r_E_**) and 1-hexene comonomer (**r_H_**) which are defined as the ratio of homopropagation to the crosspropagation rate constants. Thus, in this work, the reactivity ratios were calculated from the Fineman-Ross method and also from the Kelen-TÜdÖs method [12,13].

**Table 2:** Triad distributions of obtained copolymersdetermined by ^13^C NMR*.*

Entry	1-hexene mol^.^L^−1^	[HHH] %	[EHH] %	[EHE] %	[EEE] %	[HEH] %	[HEE] %	[H] %
1-1	0.075	0.0	23.8	13.4	15.8	10.5	36.5	37.2
1-2	0.15	0.0	21.9	15.9	19.4	10.8	32.0	37.8
1-3	0.45	19.9	32.5	9.7	4.7	18.8	14.4	62.1
1-4	0.75	36.8	33.3	4.2	0.0	15.9	9.8	74.3
1-5	1.5	28.7	37.4	3.5	0.0	14.0	16.4	72.1
2-1	0.075	0.0	11.7	18.4	28.4	7.0	34.5	30.1
2-2	0.15	7.8	14.8	18.1	21.8	13.5	24.0	40.7
2-3	0.45	16.5	28.4	13.0	6.3	18.6	17.2	57.9
2-4	0.75	48.3	11.6	7.0	13.4	19.7	0.0	66.9
2-5	1.5	43.8	34.1	0.0	0.0	12.1	9.9	78.0
3-1	0.075	4.6	10.4	18.8	28.2	10.0	27.9	33.8
3-2	0.15	8.8	20.6	17.8	11.7	15.1	26.0	47.2
3-3	0.45	29.8	24.5	9.8	5.6	13.7	16.7	64.1
3-4	0.75	27.1	25.8	10.0	6.6	15.4	15.0	63.0
3-5	1.5	31.4	32.3	4.6	2.1	11.8	17.8	68.3

**Figure 1:**
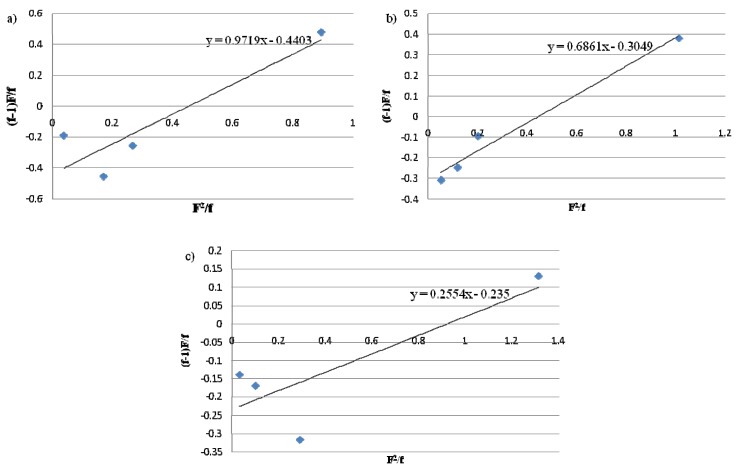
Fineman-Ross plots for the copolymers obtained with **a**) complex1 **b**) complex2 and **c**) complex3 where F = the mole ratio of ethylene and 1-hexene in the feed and f = the mole ratio of ethylene and 1-hexene in the copolymer.

**Figure 2:**
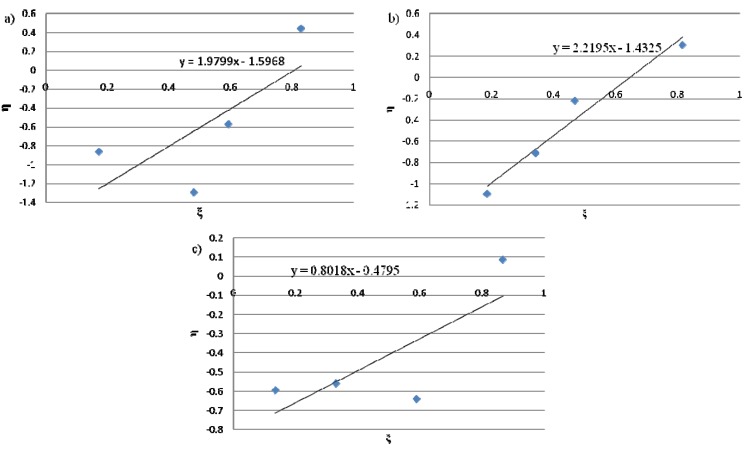
Kelen-TÜdÖs method plots for the copolymers obtained with **a**) complex 1 **b**) complex 2 and **c**) complex 3 where η = G/(α+F’), ξ = F’/(α+F’), G = (f-1)F/f, F’ = F^2^/f, α = (F’_max_F’_min_)^0.5^, F = the mole ratio of ethylene and 1-hexene in the feed and f = the mole ratio of ethylene and 1-hexene in the copolymer.

For the calculations, all copolymerizations of each series were used, except the one with the lowest concentration due to the fact it was the most affected by the experimental error. Figure 1 and Figure 2 show the Fineman-Ross and the Kelen-TÜdÖs plots for the complexes **1**, **2** and **3**, respectively, and the least squares best fit line. In general, a good fit of the experimental results in the straight line was observed in most cases. Table 3 gathers the reactivity ratios that were calculated from the mentioned methods. As seen on the Fineman-Ross and Kelen-TÜdÖs plots in Figure 1 and Figure 2, the values of r_E_ and r_H_ determined by the former fitted the experimental data obtained. Thus, a straighter line can be observed. In other words, the Fineman-Ross model was likely to give a better fit to the real data than the Kelen-TÜdÖs model. In fact, the 1-hexene reactivity ratio (r_H_) is able to describe the preference of 1-hexene incorporation into a polymer chain compared with the ethylene in the same chain end. Hence, with regard to Table 3, it can be observed that, all employed titanium complexes resulted in a tendency of ethylene incorporation into the polymer chain in comparison with 1-hexene. Furthermore, the difference in r_H_ owing to the structure of the CGC catalyst was found. The increasing ratio of 1-hexene reactivity (r_H_) along with the enhancing capability of catalyst complexes to provide higher 1-hexene content in copolymer can be concluded as follows: complex **1** > complex **2** > complex **3**. According to this result, it can be proposed that the attachment of each substituent group on ansa-fluorenyl amidodimethyltitanium complex seems to be an obstacle for higher α-olefin insertion. Overall, the values of r_E_r_H_ of the polymers obtained from all titanium complexes suggested a tendency of the formation of alternating copolymer structure (r_E_r_H_ < 1).

**Table 3:** Reactivity ratios for each copolymerization system.

Complex	r_E_^a)^	r_H_^b)^	r_E_^c)^	r_H_^d)^
1	0.97	0.44	0.76	0.30
2	0.69	0.30	0.79	0.33
3	0.26	0.24	0.32	0.10

^a)^ Ethylene reactivity ratio calculated by Fineman-Ross method; ^b)^ 1-hexene reactivity ratio calculated by Fineman-Ross method; ^c)^ Ethylene reactivity ratio calculated by Kelen-TÜdÖs method; ^d)^ 1-hexene reactivity ratio calculated by Kelen-TÜdÖs method.

## 3. Experimental

### 3.1. Materials

All operations were performed under nitrogen gas using Schlenk techniques and all solvents were dried by usual procedures and freshly distilled before use. MMAO was donated by Tosoh-Finechem Co. Ltd. Research grade ethylene (Takachiho Chemicals Co.) was purified by passing it through columns of NaOH, P_2_O_5_, and 3Å molecular sieves, followed by bubbling through a NaAlH_2_Et_2_/1,2,3,4,-tetrahydronaphthalene solution. CGC complexes were synthesized according to procedures reported previously [14,15].

### 3.2. Polymerization Procedure

Ethylene/1-hexene copolymerization was performed in a 100 mL glass reactor equipped with a magnetic stirrer. After a desired amount of 1-hexene was dissolved in a toluene solution of MMAO, copolymerization was started by adding 1 mL solution of catalyst (20 µmol). The polymerization was conducted for a certain time, and then was terminated by adding HCl/methanol solution. The obtained polymers were dried under vacuum at 333 K for 6 h.

### 3.3. Analytical Procedure

The molecular weight and molecular weight distribution were determined by GPC Waters 150 CV at 408 K using o-dichlorobenzene as a solvent and calibrated with polystyrene standards.The ^13^C-NMR spectra of copolymers were recorded at 403 K on a JEOL GX 500 spectrometer operated at 125.65 MHz in the pulse Fourier-transform mode. The samples were prepared from using 1,1,2,2-tetrachloroethane-d_2_ and the central peak of the solvent (74.47 ppm) was used as an internal reference.

## 4. Conclusions

Three CGC complexs, comprising complex **1** (unsubstituted complex), complex **2** (cyclododecyl substituent on the amine group) and complex 3 (2,7 *t*-butyl substituents on the fluorene ring), activated by MMAO were employed for ethylene/1-hexene copolymerization at 273 K to produce LLDPE. The introduction of both substituents is able to improve the activity and copolymer molecular weight. Nonetheless, it can be noticed that the 2,7 *t*-butyl group considerably impacted those features, achieving the highest activity of 3604 kg·mol^−1^ Ti·h^−1^ and the highest molecular weight of 415 kg^.^mol^−1^. The polydispersity values of all obtained copolymers are less than 2, suggesting the single site behavior of all three titanium catalyst complexes. Moreover, the 1-hexene content of copolymers can be controlled by changing the comonomer feed concentration and is dependent on the catalyst structure as well. The comonomer reactivity ratio (r_H_) result revealed that nonsubstituted titanium CGC complex yielded the highest tendency to incorporate 1-hexene into the copolymer chain.
